# Expanding Utilization of Home Dialysis: An Action Agenda From the First International Home Dialysis Roundtable

**DOI:** 10.1016/j.xkme.2021.04.004

**Published:** 2021-05-25

**Authors:** Mallika L. Mendu, José Carolino Divino-Filho, Raymond Vanholder, Sandip Mitra, Simon J. Davies, Vivekanand Jha, Kelli Collins Damron, Daniel Gallego, Michelle Seger, International Home Dialysis Roundtable Steering Committee

**Affiliations:** 1 Division of Renal Medicine, Brigham and Women’s Hospital, Harvard Medical School, Boston, MA; 2 Department of the Chief Medical Officer, Brigham and Women’s Hospital, Boston, MA; 3 Division of Renal Medicine, CLINTEC, Karolinska Institute, Campus Flemingsberg, Stockholm, Sweden; 4 Latin America Chapter (LAC-DD)-International Society for Peritoneal Dialysis; 5 Nephrology Section, Department of Internal Medicine and Pediatrics, Ghent University Hospital, Ghent; 6 European Kidney Health Alliance (EKHA), Brussels, Belgium; 7 Department of Renal Medicine, Manchester University NHS Foundation Trust, Manchester; 8 Manchester Academic Health Sciences Centre, University of Manchester, Manchester; 9 National Institute of Health Research MedTech and In-vitro Diagnostics Co-operative, Devices for Dignity, Sheffield; 10 Faculty of Medicine and Health Sciences, Keele University, Keele, United Kingdom; 11 George Institute for Global Health, University of New South Wales (UNSW), New Delhi, India; 12 School of Public Health, Imperial College, London, United Kingdom; 13 Prasanna School of Public Health, Manipal Academy of Higher Education, Manipal, India; 14 National Kidney Foundation, New York, NY; 15 European Kidney Patients Federation, Vienna, Austria; 16 Venn Strategies, Washington, DC

**Keywords:** Home dialysis, peritoneal dialysis, home hemodialysis, patient engagement, health policy, COVID-19

## Abstract

In a groundbreaking meeting, leading global kidney disease organizations came together in the fall of 2020 as an International Home Dialysis Roundtable (IHDR) to address strategies to increase access to and uptake of home dialysis, both peritoneal dialysis and home hemodialysis. This challenge has become urgent in the wake of the coronavirus disease 2019 (COVID-19) pandemic, during which patients with advanced kidney disease, who are more susceptible to viral infections and severe complications, must be able to safely physically distance at home. To boost access to home dialysis on a global scale, IHDR members committed to collaborate, through the COVID-19 public health emergency and beyond, to promote uptake of home dialysis on a broad scale. Their commitments included increasing the reach and influence of key stakeholders with policy makers, building a cooperative of advocates and champions for home dialysis, working together to increase patient engagement and empowerment, and sharing intelligence about policy, education, and other programs so that such efforts can be operationalized globally. In the spirit of international cooperation, IHDR members agreed to document, amplify, and replicate established efforts shown to improve access to home dialysis and support new policies that facilitate access through procedures, innovation, and reimbursement.

## Introduction

### Current Status of Home Dialysis Globally

Globally, 1 in 10 people have had chronic kidney disease diagnosed and 10.5 million people have advanced kidney disease, which in most cases requires treatment by either kidney transplantation or dialysis.^1^ The global number of people with advanced kidney disease is increasing at a rate of 5% to 7% per year, but 2.5 to 7 million do not have access to these life-saving treatments.^2^ Patients dependent on dialysis can either be treated in a clinic with in-center hemodialysis (HD) or at home through home HD (HHD) or peritoneal dialysis (PD).^3^^,^^4^ Though dialysis started as a largely home-based treatment in the 1960s with HHD, during the past decades it has shifted largely to in-center dialysis.^5^

Whereas overall dialysis cost is related to country income (as measured by gross domestic product per capita), home dialysis is generally less expensive than in-center dialysis in most high- and middle-income countries.^6^ Compared with in-center dialysis, home dialysis provides significant economic, quality-of-life, and clinical advantages.^7^, ^8^, ^9^, ^10^, ^11^, ^12^ Clinically, PD has been associated with better preservation of residual kidney function, fewer hospitalizations, and better quality of life, and some data suggest improved short-term survival compared with in-center HD.^13^^,^^14^ Patients receiving HHD have been demonstrated to experience important clinical advantages such as improved blood pressure, phosphate level control, and improved sleep compared with in-center patients.^15^

**Figure 1:**
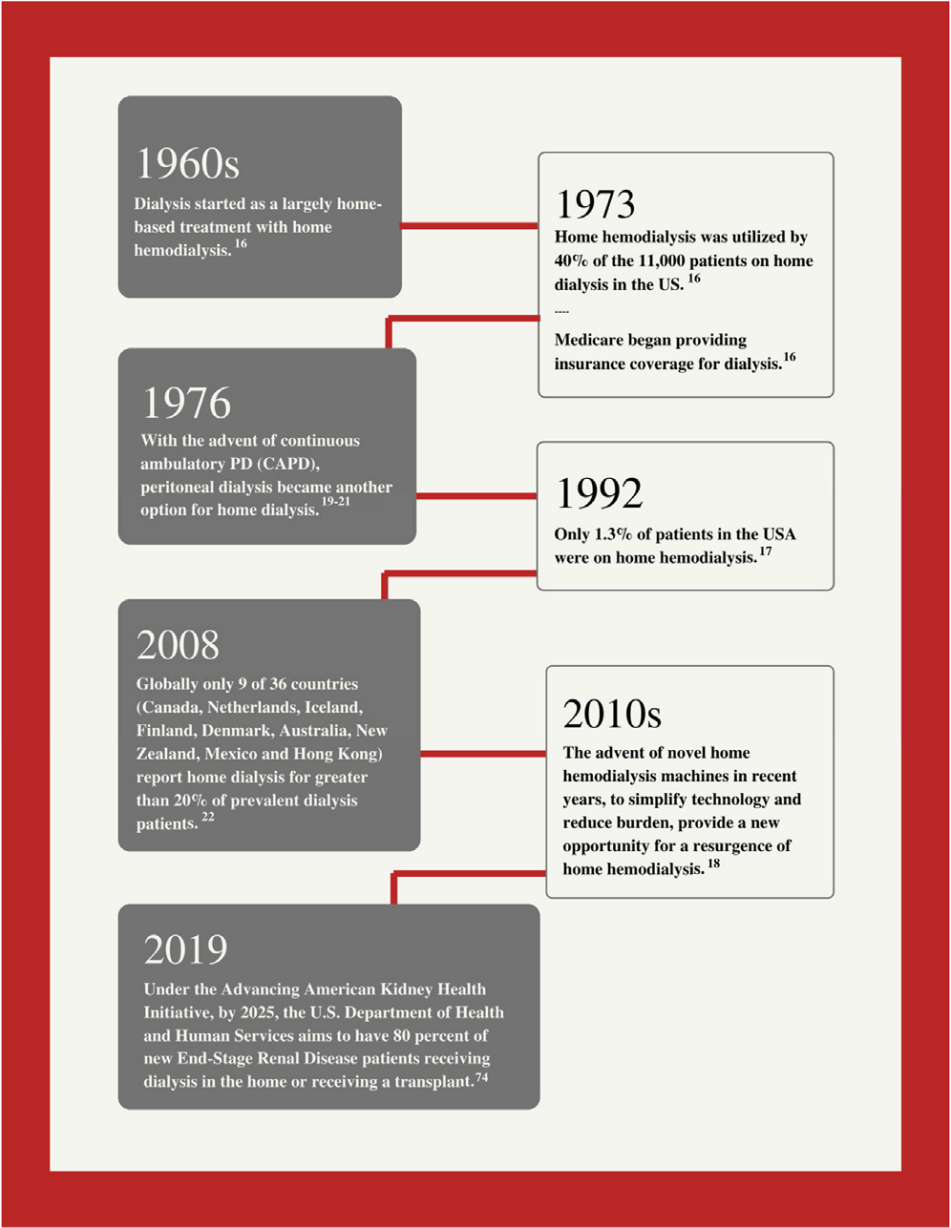
Milestones of home dialysis care delivery. Abbreviations: CAPD, continuous ambulatory peritoneal dialysis; PD, peritoneal dialysis.^16^, ^17^, ^18^, ^19^, ^20^, ^21^, ^22^, ^74^

At its inception, HHD was used by up to 40% of the 11,000 patients receiving HD in the United States, but the passage of the Social Security Amendments of 1972, which included an entitlement for dialysis patients, changed this paradigm in favor of in-center HD. Although the Medicare End-Stage Renal Disease program greatly increased the number of patients able to access dialysis treatment overall, it created financial incentives for the growth of large dialysis organizations with economies of scale to establish in-center facilities. Subsequently care shifted from HHD to in-center HD due to these unintended disincentives to home dialysis, and by 1978, <15% of patients dialyzed at home.^16^ By 1992, only 1.3% of patients in the United States were receiving HHD.^17^ We present a summary of key milestones in home dialysis care delivery in Fig 1.

However, the advent of novel HHD machines in recent years, to simplify technology and reduce burden, provide a new opportunity for a resurgence of HHD.^18^ Different therapeutic modalities within HHD, such as low-flow dialysates with bags versus conventional monitors and much-needed efforts to use tunneled central venous catheters if no other vascular access option is possible, are also important when considering how to increase access to HHD. Further, with the advent of continuous ambulatory PD in 1976, PD became another option for home dialysis because all patients receiving continuous ambulatory PD or automated PD can be treated at home.^19^, ^20^, ^21^ Globally only 9 of 36 countries (Canada, Netherlands, Iceland, Finland, Denmark, Australia, New Zealand, Mexico, and Hong Kong) report home dialysis for >20% of prevalent dialysis patients.^22^ In addition, it is worth noting that differences in home dialysis uptake across centers have been observed even in countries for which overall prevalence is low; localities with a high penetration of home dialysis should be considered when thinking about how to overcome country-wide logistical, educational, or financial barriers.

### The COVID-19 Pandemic and Home Dialysis

During the past few decades, many kidney health organizations globally have strongly advocated for policy and practice changes to increase access to and uptake of home dialysis. These efforts have seen varying levels of success over the years. The coronavirus disease 2019 (COVID-19) pandemic has further emphasized the importance of enabling greater numbers of patients to perform dialysis at home, given that dialysis patients are at high risk for developing severe COVID-19 outcomes compared with the general population and treatment in a dialysis center challenges efforts to physically distance.^23^ Home therapies are recognized as ideal when pandemics break out, particularly compared with in-center dialysis in which patients must visit a health care facility at least 3 times per week.^24^ Recent Canadian and Italian data demonstrated that patients receiving in-center dialysis were up to 3 times more likely to test positive for COVID-19 than patients receiving home dialysis.^25^^,^^26^ The US Renal Data System recently reported that the COVID-19 hospitalization rate for patients undergoing in-center HD was 3 to 4 times that of patients receiving PD and HHD.^27^^,^^28^ These observations have prompted a sense of urgency among clinicians and advocates to find immediate ways to make home dialysis more available for patients with kidney disease. The concerted push to help patients access home dialysis during the pandemic has shed light on many long-standing obstacles to home dialysis access that limit uptake.

Given the critical juncture of the COVID-19 pandemic, vulnerability of the kidney disease population, and long-standing demonstrated advantages of home dialysis modalities, leading kidney health organizations throughout the international community formed an International Home Dialysis Roundtable (IHDR). The IHDR met during a 2-part virtual meeting (October and November 2020) to consider practical steps for increasing access to home dialysis, both during the still raging pandemic and beyond (meeting description and invitation provided in Items S1 and S2). The meetings were divided into sections identifying specific challenges and designing action solutions for barriers to home dialysis related to clinicians, patients, and institutions (including governments) and innovation challenges. The leaders who participated in this landmark conference committed to work independently through their organizations and collaboratively as a group to address barriers that currently limit home dialysis uptake by focusing on 4 clear domains of action opportunities: (1) establishment of international standards to increase home dialysis; (2) empowerment of patients, improved shared decision making, and reduction of paternalism among health care providers; (3) creation of a “culture of support” for home dialysis among health care providers through education and training; and (4) creation of internationally shareable best practices on successful strategies to reduce institutional or governmental roadblocks to home dialysis (Item S3). Each participant organization committed to use their own association’s upcoming meetings and online platforms to advance best practices and foster further discussion of these goals, to better equip colleagues in disparate regions, find and develop home dialysis patient and clinician “champions,” and advocate for pro-home dialysis policy change in their respective regions.

In this article, we share highlights of the IHDR discussion and outline the action recommendations (Box 1)^29^, ^30^, ^31^, ^32^, ^33^, ^34^ agreed on by its members that can serve as a road map for patients, providers, institutions, and policy makers to promote home dialysis. We tasked membership and participants to engage with their organizations to operationalize action items in a resource- and context-specific manner in different geographies.

Box 1 **International Home Dialysis Roundtable Policy Action Plan**

**Table:** 

**Action Items**
I**nternational Standards and Cooperation** Action recommendations were centered around home dialysis education, training, and advocacy standardization across countries: 1) Development of centralized centers of excellence in home dialysis throughout both developing and developed countries, 2) Standardization of home dialysis education and training for clinicians and health care professionals working with nephrology and dialysis, 3) Alignment and regional tailoring of advocacy messages through patients and caregivers to motivate policy makers to action in favor of home dialysis strategies, and 4) Educational and comprehensive education programs about needs of patients and caregivers to reduce the burden of dialysis.
**Patient Empowerment and Education** Action recommendations focused on supporting and amplifying existing broad-based patient education efforts and establishing new avenues for creating awareness: 1) Establishment of kidney disease awareness campaigns through social media and patient-centered events in collaboration with patient-led organizations; 2) Development and delivery of country-specific home dialysis training modules to clinicians and patients, such as the International Society for Peritoneal Dialysis and International Society for Hemodialysis’s current educational materials^29^; 3) Focus on patient stories on a local, national, and regional basis for use in advocacy, provider education and patient awareness programs; 4) Commitment to work together to create and promote in-person and virtual patient education programs; and 5) Involvement of patients in home dialysis education for patients and clinicians.
**Culture of Support for Home Dialysis** Action recommendations focused on developing and sharing advocacy and educational programs for home dialysis clinicians, across global organizations: 1) Creation of programs across global kidney disease organizations, such as the World Kidney Disease Games, International Congress of Nephrology, International Society of Nephrology, and organizations focused on related conditions, such as diabetes, cardiovascular disease and cancer, to advocate across specialties for policy and practice changes related to home dialysis; 2) Development of clinician training programs for certain key procedures related to dialysis initiation (eg, the South African and Latin American peritoneal dialysis catheter implantation training programs)^30^, ^31^, ^32^; 3) Expansion of an international webinar program aimed at educating clinicians about home dialysis, including one that the International Society of Peritoneal Dialysis is currently leading^29^; 4) Creation of regional clinician mentoring programs, such as Project ECHO in the United States, and the International Society of Nephrology sister center programs^33^^,^^34^; and 5) Involvement of home dialysis patients in all these actions because their own home dialysis experience is conveyed directly to other patients, to clinicians, and to policy makers.
**Mitigation of Institutional or Governmental Roadblocks** Action recommendations focused on engaging policy leaders in varied countries to close the gap between in-center and home dialysis reimbursement: 1) Creation of country-wide best practices on how to advocate with policymakers to ensure that vascular access, peritoneal dialysis catheter placement, and home hemodialysis training programs are prioritized; 2) Incentivized peritoneal dialysis catheter training programs to promote catheter placement; 3) Research efforts that seek to determine how payment policy affects uptake and which barriers to home dialysis are unrelated and how changes to government policy could affect home dialysis uptake levels; and 4) Involvement and engagement of health care leaders, such as executives of health systems, health policy experts, and health care organization leaders.

## International Standards and Cooperation

Differences in culture, infrastructure, payment policy, and economic status exist among countries and regions, particularly among high-, middle-, and low-income countries, but the COVID-19 pandemic has highlighted that many barriers to home dialysis are shared across the world. Some of these challenges include adequate provider, clinician, and patient education; access to telehealth technologies; a lack of home dialysis “champions”; and a need to further empower and train patients and their caregivers to take an active role in their care.^35^, ^36^, ^37^ Specific to the COVID-19 pandemic, some kidney disease leaders expressed concern that physicians were unable to conduct certain procedures required for the initiation of home dialysis, particularly PD due to regulatory constraints related to prioritization of catheter procedures.^38^ As a consequence, candidates for PD are often forced to either opt for in-center HD or postpone the start of a necessary treatment. However, in other countries with no such regulatory constraints, PD demand and use increased during this period.^39^ Whereas some organizations, including many of those in attendance at the IHDR, work cross-nationally and/or cross-regionally to overcome the challenges mentioned and have seen important successes, all participants committed to a more strategic and intentional partnership to “move the needle” on home dialysis rates and agreed that streamlining of disparate initiatives is needed.

### Action Recommendations

Action recommendations were centered around home dialysis education, training, and advocacy standardization across countries.

1) Development of centralized centers of excellence in home dialysis throughout both developing and developed countries,
2) Standardization of home dialysis education and training for clinicians and health care professionals working with nephrology and dialysis,
3) Alignment and regional tailoring of advocacy messages through patients and caregivers to motivate policy makers to action in favor of home dialysis strategies, and
4) Comprehensive education programs about needs of patients and caregivers to reduce the burden of dialysis.

## Patient Empowerment and Education

It is widely accepted that a lack of adequate patient and caregiver education is one of many factors that have led to the low rates of home dialysis uptake globally, with most patients receiving in-center dialysis.^35^^,^^40^ Besides physicians and nurses who train and take care of home dialysis patients, there is a lack of physician knowledge related to home dialysis, with very few medical professionals having ever seen a patient performing HD or PD at home.^41^ Compounding this challenge is the paternalistic attitude of some hospitals and clinicians, assuming limited patient understanding about dialysis and that patients cannot effectively dialyze at home.^42^ This often results in clinicians steering patients toward in-center dialysis even if they are potential candidates for home dialysis.^42^ Despite evidence that shared decision making between nephrologists and patients allows for mutual agreement, a survey study of nephrologists demonstrated that there are varied approaches to decision making related to dialysis.^43^

Another issue is the phenomenon of “crashing into dialysis,” in which patients are referred late to the nephrology clinic when they are in imminent need of dialysis.^44^ Subsequently, they begin dialysis in-hospital, without education about dialysis modality options, and as a result, the opportunity for home dialysis is missed.^45^ This tendency has been compounded by the COVID-19 pandemic.^46^ Even if a patient does not “crash” into dialysis, they are often not provided timely predialysis education, forestalling proper information and empowerment for decision making, including modality choice.^47^ Some experts have advocated for commencement of predialysis education based on kidney function level or symptoms, a widely accepted marker for education initiation is a glomerular filtration rate < 20 to 25 mL/min/1.73 m^2^.^48^

Further, research has shown that adequate predialysis education can reduce these crash starts and increase the likelihood of choosing home dialysis or another self-care modality.^48^ Self-care modalities include the self-care dialysis unit, another area that would benefit from additional research and exploration. Self-care and transitional dialysis units, along with innovative devices, are examples of new pathways and technologies, which when supported by appropriate policies and reimbursement could drive resurgence in home therapies. IHDR attendees agreed to work through their organizations and with each other to empower patients and caregivers to participate actively in choosing the best modality for their medical needs and for the patients already dependent on home dialysis to share their stories during advocacy efforts in favor of home dialysis.

During the IHDR sessions, testimony was provided by patients about the barriers they faced in receiving home dialysis. One patient testified: “There should be fully transparent discussions with patients on ALL options available. Many patients have never received a full briefing in advance of their options, which is unacceptable.” Other patients expressed their support for home dialysis therapy, stating that it allowed them to maintain a high level of independence and the ability to work and travel. Moreover, home dialysis has facilitated physical distancing during the pandemic.^49^ One HHD patient stated that the first barrier to be tackled is that “home dialysis is not available for everyone” and that it would be helpful to develop a tool for decision making. Another patient receiving HHD for many years emphasized the need for autonomy to determine their own treatment at home.

There are countries in which patients are not allowed to perform HD at home on their own and a trained care partner (or in some cases a nurse and/or a nephrologist) must be present during the entire session. Some countries do not support professional-assisted home dialysis, and this can be a barrier for those without a care partner if unable to perform the treatment alone.^50^ The importance of supporting patients unable to carry out home dialysis alone, facilitating assisted home dialysis, either with the help of a relative, a caregiver or a health care professional, was discussed. Assisted home dialysis is a meaningful option to home care; dedicated actions by different groups (such as health care professionals, patients with kidney disease, caregivers, community, and payors) should be considered.^51^ There were concerns raised about managing, storing, organizing, and disposing of dialysis supplies at home and the need for investment in resources to better support patients and families. All these patient testimonies reflect a desire for increased clinician efforts to empower patients to take charge of their health decision making and dialysis journey. Increased access to conversations on self-management of kidney disease, the rationale behind why certain restrictions or requirements are put in place, and how to regain what some studies have termed “lost vitality” are key.^52^

Patient stakeholders in the IHDR sessions saw a role for patients as part of the home dialysis education and training process for both patients and clinicians. Although many patients noted that they had supported other patients either through formal peer support programs or informally through personal connections or within their clinic, they believed that patient expertise has been grossly underused. They noted significant room for growth in systematizing patient partnership at all levels of patient and clinician education and identified many missed opportunities for patients to partner both with individual providers, dialysis centers, hospitals, and health care systems. Patients’ unique perspectives would serve to dispel myths and demonstrate the benefits and possibilities that a home dialysis lifestyle can afford, both normalizing and revolutionizing the shared understanding of the home dialysis patient experience.

Clinicians remarked on the important role of expanded telemedicine care during the pandemic that has facilitated remote care of home dialysis patients, and as a result, efforts are needed to mitigate the digital divide affecting vulnerable patient populations.^53^^,^^54^

### Action Recommendations

Action recommendations focused on supporting and amplifying existing broad-based patient education efforts and establishing new avenues for creating awareness:

1) Establishment of kidney disease awareness campaigns through social media and patient-centered events in collaboration with patient-led organizations;
2) Development and delivery of country-specific home dialysis training modules to clinicians and patients, such as the International Society for PD and International Society for HD’s current educational materials^29^;
3) Focus on patient stories on a local, national, and regional basis for use in advocacy, provider education and patient awareness programs;
4) Commitment to work together to create and promote in-person and virtual patient education programs; and
5) Involvement of patients in home dialysis education for patients and clinicians.

## Culture of Support for Home Dialysis

Clinical champions for home dialysis have made critical differences in the uptake of home therapy in various countries.^55^ Nurses in particular are crucial in the administration of and training for home dialysis and they have a unique active connection to dialysis patients, with whom they often have more personalized conversations than physicians.^56^^,^^57^^,^Although the kidney disease community can benefit from identifying and promoting additional strong clinical leaders to educate the public and policymakers about home dialysis, clinician advocates are not present in every region or health system.^58^ IHDR participants discussed the importance of a wholesale shift in clinical culture to reshuffle the current philosophy of prioritizing treatment options, moving from a system that directs most patients to in-center dialysis to one that more broadly recognizes and supports home dialysis as a viable first-choice option for patients. There was a recognition that a coordinated effort must be made to increase the number of nurse and other clinical champions for home dialysis, and any cultural shift toward home dialysis in the nephrology community must include nurses and the organizations that represent them as well.

In addition, medical, nursing, and pharmacy students should be thoroughly and objectively educated about home dialysis therapies. Furthermore, the role of home dialysis should be an important part of clinical fellowship curricula in not only nephrology, but also clinicians managing patients with kidney failure, such as general practitioners, family physicians, cardiologists, diabetologists, oncologists, or vascular surgeons.^59^ This should be followed by opportunities to further educate clinicians throughout their careers, mentoring for clinicians, and promoting the application of shared decision making tools for clinicians and patients to use. Finally, an important takeaway from the IHDR discussions was that many organizations were not aware of existing curriculum and advocacy programs led by other organizations and that sharing efforts could further help advance a culture shift.

### Action Recommendations

Action recommendations focused on developing and sharing advocacy and educational programs for home dialysis clinicians, across global organizations:

1) Creation of programs across global kidney disease organizations, such as the World Kidney Disease Games, International Congress of Nephrology, International Society of Nephrology, ands organizations focused on related conditions, such as diabetes, cardiovascular disease, and cancer, to advocate across specialties for policy and practice changes related to home dialysis;
2) Development of clinician training programs for certain key procedures related to dialysis initiation (eg, the South African and Latin American PD catheter implantation training programs)^30^, ^31^, ^32^;
3) Expansion of an international webinar program aimed at educating clinicians about home dialysis, including one that the International Society of PD is currently leading^29^;
4) Creation of regional clinician mentoring programs, such as Project ECHO in the United States, and the International Society of Nephrology sister center programs^33^^,^^34^; and
5) Involvement of home dialysis patients in all these actions because their own home dialysis experience is conveyed directly to other patients, to clinicians, and to policy makers.

## Mitigation of Institutional or Governmental Roadblocks to Home Dialysis

Two major practical barriers to home dialysis use include vascular or peritoneal catheter placement and payment disparity (between in-center and home dialysis modalities), which are largely institutional or governmental in origin.^60^

Limited access to the procedures to place the catheters needed to enable PD has long been a challenge in many countries. The limitations stem from many factors, including the need for more and better training on the procedure, lack of diversity in insertion methods, and challenges related to access to operating rooms that may be in short supply.^61^, ^62^, ^63^ The COVID-19 pandemic may have exacerbated these challenges as the need for immediate catheter placement has become acute for larger numbers of patients who could benefit from starting home dialysis to physically distance safely.^39^ An additional major barrier to HHD use is the amount of upfront cost needed for facilities to ensure necessary staffing and infrastructure to train patients.^64^^,^^65^

Governmental dialysis reimbursement systems are typically designed to control costs while also ensuring high quality of treatment and health outcomes. Even so, many health systems are constructed in a way that directs patients to in-center dialysis rather than more economically beneficial home dialysis.^66^^,^^67^ An important evolution in payment reform took place in the United States in 2011 when equal reimbursement of all dialysis modalities was implemented and resulted in greater PD uptake (9.4% to 12.6% early PD initiation and 12.1% to 16.1% late PD use).^68^

However, there is an unclear relationship between reimbursement and PD uptake in other countries. For example, the highest rate of PD use in the European Union is in Scandinavia (>28%), despite lower reimbursement than for in-hospital HD.^69^^,^^70^ However, these governments reimburse real costs and not a flat sum, obviating financial benefit or loss to the clinical team. In addition, there are educational “home dialysis first initiatives” led by national or local kidney health communities.^71^ Some data suggest that equity of reimbursement coupled with a PD-first policy may be effective in promoting greater PD use.^71^ HHD reimbursement is often intermediate between in-center HD and PD.^72^ However, most countries have no specific regulations for HHD, which can be counterproductive for the uptake of this strategic option.^73^ Finally, further research and engagement of policy makers is warranted regarding whether incentivizing home dialysis beyond in-center rates would increase uptake for PD and/or HHD.

The IHDR discussion focused on that fundamental changes are needed to the structure of medical payments and reimbursements that support vascular or peritoneal access placement and home dialysis modalities. Discussants advocated for parity in reimbursements among the various dialysis modalities, coupled with a home dialysis-first approach supported by governments and their health systems.

### Action Recommendations

Action recommendations focused on engaging policy leaders in varied countries to close the gap between in-center and home dialysis reimbursement:

1) Creation of country-wide best practices on how to advocate with policy makers to ensure that vascular access, PD catheter placement, and HHD training programs are prioritized;
2) Incentivized PD catheter training programs to promote catheter placement;
3) Research efforts that seek to determine how payment policy affects uptake and which barriers to home dialysis are unrelated and how changes to government policy could affect home dialysis uptake levels; and
4) Involvement and engagement of health care leaders, such as executives of health systems, health policy experts, and health care organization leaders.

## Conclusions

The IHDR members agreed that these discussions should be a springboard for new relationships and strategic partnerships between patients, experts, organizations, and advocates from around the world that can be leveraged to increase access to home dialysis. Discussants expressed a desire to increase and expand work across countries and regions to address limitations to home dialysis and to collaborate to ensure that successful projects can be replicated from one region to another. A total of 91% of participating organizations reported that they are working on a project relevant to increasing home dialysis uptake in their home country, region, or constituency that they would be willing to share with the rest of the group. We have asked members to share updates from their organizations as they make progress toward these articulated goals, and we have proposed a follow-up meeting in 2021 (Box 2).

Box 2 **IHDR Summary of Operationalizing Action Items**

We intend to continue our work with the IHDR together as a group and within each individual organization that signed the consensus statement to increase home dialysis globally. Specifically:

**Current**

Each organization has committed to work on the manuscript’s action items within their individual structures, programming, and advocacy work. We expect to see updates from these organizations as they make progress toward their individual goals.

**Quarter 2, 2021**

We anticipate conducting a survey of IHDR Steering Committee members and attendees to monitor each group’s progress toward individual goals. We would also conduct a call at this time to disseminate updates and ensure that we are working together as appropriate.

**Quarter 4, 2021**

We have proposed a meeting of all stakeholders.

Abbreviation: IHDR, International Home Dialysis Roundtable.

There was broad consensus that the action recommendations be shared with government policy regulators and administrators as policy changes are needed to affect home dialysis uptake around the world. The IHDR participants agreed that the kidney community must use the enthusiasm from the roundtable discussion and the momentum created by the pandemic to support and elevate home dialysis initiatives worldwide. Discussants made it clear that they are eager to act on the commitments they made in the consensus statement and strengthen their relationships globally to promote home dialysis.

## Supplementary Material

Supplementary File (PDF) Items S1-S3
