# Supplementary material for: Expanding Utilization of Home Dialysis: An Action Agenda From the First International Home Dialysis Roundtable
Source: Kidney Med. 2021 May 25;3(4):635–43. doi: 10.1016/j.xkme.2021.04.004 (PMC8350829; doi:10.1016/j.xkme.2021.04.004)
Supplement: Supplementary File (PDF) — Items S1-S3 [file mmc1.pdf]

Item S1: Recap of International Home Dialysis Roundtable Events

1A: First International Home Dialysis Roundtable

The first of two virtual IHDR conferences was held on October 1, 2020. Nearly 60 experts in home dialysis and renal care, representing most of the largest and most prominent international kidney disease societies, participated to review current practices and recent studies examining home dialysis, share new insights from treating dialysis patients in the context of the COVID-19 pandemic, and discuss new ways to improve access to home dialysis. Discussions centered on lessons learned during the pandemic and how those learnings can be applied to overcome barriers to home dialysis and affect policy change for the future.

Five presentations and discussions were featured:

- **Clinician Barriers: Education, Training & Institutional Culture**
    - Presenters: Dr. Alp Ikizler and Dr. Mallika Mendu, American Society of Nephrology
- **Patient Barriers**
    - Presenters: Daniel Gallego, European Kidney Patient Federation and Kelli Collins, (US) National Kidney Foundation
- **Regulatory & Payment Policy Barriers to Innovation and Implementation**
    - Presenters: Dr. Raymond Vanholder, European Kidney Health Alliance, Dr. Paul Komenda, Quanta Dialysis Technologies, and Dr. Angelito Bernardo, Baxter
- **Regional Variances in Home Dialysis Access & Uptake**
    - Presenters: Dr. José Carolino Divino-Filho, International Society of Peritoneal Dialysis- Latin America Chapter, and Dr. Haruki Wakai, Asian Pacific Society of Nephrology
- **Policy & Practice Solutions: How Do We Shift the Landscape?**
    - Discussion Leaders: Dr. Vivekanand Jha, International Society of Nephrology and Dr. Simon Davies

1B: Second International Home Dialysis Roundtable

The second meeting of the International Home Dialysis Roundtable was held on November 17, 2020 and brought forward specific recommendations of the types of

actions that stakeholders can take to remove current challenges to home dialysis. The event again featured input from critical leaders in home dialysis from major kidney disease organizations around the world. In all, 64 attendees joined the leadership dialogue.

The event was split into three moderated 40-minute discussion sessions:

- **Actions to overcome clinician obstacles to home dialysis**
    - Moderator: Dr. Vivekanand Jha, International Society for Nephrology
    - Discussants: Ryan Murray and David White, American Society of Nephrology, Dr. Ali Abu-Alfa, American University of Beirut, Daniel Gallego, European Kidney Patient Federation, Dr. Raymond Vanholder, European Kidney Health Alliance, Dr. Helene Boisvert, Fondation du Rein, Nieltje Gedney, Home Dialyzors United, Dr. Edwina Brown, Imperial College Healthcare NHS Trust, Dr. Sharon Nessim, International Society of Peritoneal Dialysis, Colin White, Irish Kidney Foundation, Dr. Simon Davies, Dr. Brett Cullis, Life Hilton Hospital, Anthony Gucciardo, National Kidney Foundation, Dr. Thyago Proenca de Moraes, Pontificia Universidade Catolica do Parana, and Henrik Eriksson, Swedish Kidney Foundation
- **Actions to overcome patient obstacles to home dialysis**
    - Moderator: Kelli Collins, National Kidney Foundation
    - Discussants: Erich Ditschman, National Kidney Federation, Daniel Gallego, European Kidney Patients Federation, Nieltje Gedney, Home Dialyzors United, Colin White, Irish Kidney Association, Robert Terzan, United Kidney Patients Association of Slovenia, and Henrik Eriksson, Swedish Kidney Foundation
- **Actions to overcome obstacles related to innovation and implementation**
    - Moderators: Dr. Raymond Vanholder, European Kidney Health Alliance and Dr. Angelito Bernardo, Baxter
    - Discussants: Kelli Lester, Baxter, Dr. José Carolino Divino-Filho, International Society of Peritoneal Dialysis-Latin America Chapter, Dr. Simon Davies, Dr. Thyago Proenca de Moraes, Pontificia Universidade Catolica do Parana, Fokko Wieringa, Imec and Dutch Kidney Foundation, Dr. Edwina Brown, Imperial College Healthcare NHS Trust, Dr. Vivekhanand Jha, International Society of Nephrology, Daniel Gallego, European Kidney Patients Federation, Robert Terzan, United Kidney Patients Association of Slovenia, Dr. Alp Ikizler, American Society of Nephrology, Dr. Talerngsak Kanjanabuch, Chulalongkorn University, Dr. Brett Cullis, Life Hilton Hospital, and Nieltje Gedney, Home Dialyzors United

Item S2: Invitations for the International Home Dialysis Roundtable

International Home
Dialysis Roundtable

Advancing Home Dialysis:
The Imperative from the Pandemic and Beyond (Part I)

Across the world, the COVID-19 pandemic has made clear the urgency of ensuring equitable access to home dialysis so that kidney disease patients can stay safe at home. Advocacy groups from around the globe will gather virtually to develop policy recommendations for access and uptake of home dialysis-during a global pandemic. We invite you to join us for the first of two roundtables as a discussant alongside Steering Committee members from leading advocacy organizations around the world for a dialogue about what can be done, now and beyond the pandemic, to meet this vital goal for ESRD patients.

There will be a second follow-on roundtable to discuss practical and policy initiatives that align with the barriers and goals identified here. Watch for that invitation shortly!

Thursday, October 1, 2020

7:00 - 10:30 AM EDT

Register Here

Steering Committee Members

American Society of Nephrology • Asia Pacific Society of Nephrology • Baxter International • Euro PD • European Kidney Health Alliance • European Kidney Patient Federation • European Renal Association - European Dialysis and Transplant Association • International Society for Peritoneal Dialysis • International Society of Nephrology • National Kidney Foundation • Quanta

Supported with resources from Baxter

International Home Dialysis Roundtable

Advancing Home Dialysis: The Imperative from the Pandemic and Beyond (Part II)

Advocacy groups from around the globe gathered virtually for Part I of the Roundtable series to develop policy recommendations for access and uptake of home dialysis-during a global pandemic. The follow-on roundtable will continue the conversation around practical and policy initiatives, building on the important work of Roundtable 1, where participants and presenters highlighted barriers to and goals for increasing access to home dialysis worldwide.

We invite you to join us for the next roundtable in the series as a discussant alongside Steering Committee members from leading advocacy organizations around the world for a dialogue about what can be done, now and beyond the pandemic, to meet this vital goal for ESRD patients.

Tuesday, November 17, 2020

7:30 - 10:00 AM EST

Register Here

Steering Committee Members

American Society of Nephrology • Asia Pacific Society of Nephrology • Baxter International • Euro PD • European Kidney Health Alliance • European Kidney Patient Federation • European Renal Association - European Dialysis and Transplant Association • International Society for Peritoneal Dialysis • International Society of Nephrology • National Kidney Foundation • Quanta Dialysis Technologies

Supported with resources from Baxter

Item S3: Consensus Statement on Increasing Home Dialysis

The undersigned members of the international kidney disease community commit to work independently and together throughout the COVID- 19 pandemic and after it has ended to address and remove barriers to home dialysis access and increase global uptake rates. We believe that it is our obligation to help facilitate access to home dialysis for all eligible kidney failure patients and understand that true change can only be made when we stand together and work as one global community. Specifically, we pledge to work independently and together to:

- Increase the reach and influence of key stakeholders and spread messages of hope related to home dialysis;
- Empower patients, institutionalize shared-decision-making and reduce so-called paternalism in nephrology, leading to increased patient involvement in modality choice;
- Cultivate a clinical culture of support for home dialysis, including opportunities for education; and
- Share best practices to reduce system-level (institutional or governmental) barriers to home dialysis.

Signed,
American Society of Nephrology
Asian Pacific Society of Nephrology
Baxter Healthcare
European Kidney Health Alliance
European Kidney Patient Federation
Dr. Simon Davies
Dr. Sandip Mitra International Society for Peritoneal Dialysis-Latin America Chapter
International Society of Nephrology
National Kidney Foundation
Quanta Dialysis Technologies
